# Implementing patient and public involvement (PPI) in eye research: reflections from developing a research study on Geographic Atrophy treatment acceptability

**DOI:** 10.1186/s40900-025-00747-7

**Published:** 2025-08-01

**Authors:** Jamie Enoch, David Matthews, Arevik Ghulakhszian, Mandeep Sekhon, Tamsin Callaghan, David Crabb, Christiana Dinah, Deanna Taylor

**Affiliations:** 1 Department of Optometry and Visual Sciences, School of Health and Psychological Sciences, City St George’s, University of London, London, UK; 2 Patient Advisor, London, UK; 3 Ophthalmology Department, Central Middlesex Hospital, London North West Healthcare NHS Trust, London, UK; 4 Population Health Research Institute, City St George’s, University of London, London, UK; 5 Research and Development Team, Royal Free London NHS Foundation Trust, London, UK; 6 Department of Brain Sciences, Imperial College London, London, UK

## Abstract

**Background:**

Awareness of the importance of patient and public involvement (PPI) in ophthalmology research is growing, ensuring studies align with patient priorities and experiences. However, there is limited literature exploring the practicalities and details of how PPI may be conducted within this field. In this case study of PPI within an ophthalmological research project, we aim to provide a transparent, in-depth illustration of how PPI was implemented and helped to shape the Acceptability of Geographic Atrophy INjections (AGAIN) study. The AGAIN study is focused on patients’ perspectives regarding the acceptability of new intravitreal (eye) injection treatments for Geographic Atrophy, an advanced form of age-related macular degeneration.

**Main text:**

This commentary explores how PPI was undertaken to shape the design of the two work packages of the AGAIN study. In work package 1, the AGAIN pilot, we worked with a group of patient advisors to design materials for a mixed-methods questionnaire. This questionnaire consisted of Likert-type scale questions, semi-structured interview questions, and an elicitation task considering different hypothetical treatment scenarios. Eight patient advisors provided their input into the design of this questionnaire, and we discuss examples of the concrete changes to the research materials based on the advisors’ feedback. In work package 2, we carried out several rounds of consultation with patient advisors to develop a pre-validated quantitative questionnaire on Geographic Atrophy treatment acceptability. This involved using ‘think-aloud’ techniques to explore the questionnaire’s validity, clarity, and comprehensibility. We discuss some of the challenges that may arise when taking on board divergent points of view, and how to maximise comprehensibility without compromising fidelity to a validated questionnaire.

**Conclusions:**

Our experience attests to the importance of listening to the insights of patients and those with lived experience in the early stages of designing research, while also ensuring that PPI remains continually integrated throughout the study lifecycle. Our PPI approach evolved in an ad-hoc fashion, and we suggest that given its beneficial impact for our study, PPI should be carefully planned for and adequately resourced in patient-centred ophthalmological research programmes.

**Supplementary Information:**

The online version contains supplementary material available at 10.1186/s40900-025-00747-7.

## Abstract (plain language summary)

This article highlights the importance of involving patients and the public in eye health research, ensuring research addresses their concerns and experiences. In this article, we reflect on our experience of patient involvement, and the lessons we learned. We present a case study of how we involved patients as advisors on our study, the Acceptability of Geographic Atrophy INjections (AGAIN) study. The AGAIN study is exploring patient views on new eye injection treatments for Geographic Atrophy, a later stage form of age-related macular degeneration, which can cause sight loss among older adults.

Our article is organised around the two different parts of the AGAIN study. In the first part, we reflect on how we worked with a group of patient advisors, who were people living with Geographic Atrophy, to design a study using surveys and interviews to understand views on Geographic Atrophy treatments. In the second part, we reflect in detail on working with patient advisors to design and refine a more formal questionnaire. This questionnaire aimed to assess how acceptable people living with Geographic Atrophy consider the treatments to be. In both parts of the AGAIN study, the patient advisors provided feedback that helped to improve the clarity and relevance of the questionnaire. Our article illustrates the value of patient input into eye health research. We also advocate involving patients from the start of research projects, and considering methods to balance conflicting viewpoints.

**Supplementary Information:**

The online version contains supplementary material available at 10.1186/s40900-025-00747-7.

## Introduction

Patient and public involvement (PPI) in health research can be conceptualised as research “carried out ‘with’ or ‘by’ members of the public rather than ‘to’, ‘about’ or ‘for’ them” [28]. Patient and public involvement is vital throughout the lifecycle of research projects and can be particularly important in the “ideas phase” (NIHR [27]) of the research where patients and members of the public have an opportunity to shape and influence the direction of research before protocols become established. As in other fields, there has been a culture shift in ophthalmology research, recognising the importance of the perspectives of patients and members of the public in shaping the research agenda [11, 37]. With a clear understanding of *why* PPI is important, the focus has shifted to more nuanced discussion of *how* meaningful involvement can take place and where aspects of the involvement process can be improved [10].

Therefore, in this commentary, we explore the *how* of involvement by reflecting on PPI in the context of the Acceptability of Geographic Atrophy INjections (AGAIN) study. After briefly providing background context about the AGAIN study, we will consider the process of PPI – particularly in the design phase—for both the mixed-methods study and the quantitative questionnaire study. We will then reflect in detail on the process of PPI in developing and adapting a pre-validated questionnaire^1^; in particular, we will consider how the perspectives of patients and the public can be heard while also balancing this against the perceived need to remain faithful to a pre-validated questionnaire instrument. We also consider the challenge of ensuring all voices are heard while trying to arrive at some kind of consensus.

Although this is a commentary rather than formal evaluation of PPI, we have been guided by the Guidance for Reporting Involvement of Patients and Public (GRIPP) 2 principles [40] as we reflect on how PPI has been integrated into the AGAIN study (Appendix 1).

## The AGAIN study

The AGAIN study aims to explore the acceptability of new intravitreal (eye) injection treatments for Geographic Atrophy (GA), an advanced form of age-related macular degeneration (AMD). Geographic atrophy is a significant cause of sight loss globally, accounting for 26% of legal blindness in the United Kingdom [32]. Complement inhibitors, delivered by regular intravitreal injections in the eye, have recently been demonstrated to slow down progression of GA lesions in phase 3 trials [12, 16, 18]. Since embarking on the AGAIN study, in 2023 the US Food and Drug Administration approved two intravitreal treatments for GA, namely SYFOVRE (pegcetacoplan) and IZERVAY (avacincaptad pegol). However, concerns around clinically meaningful functional benefits and increased risks of developing wet (neovascular) AMD due to the GA treatments have contributed to lack of approval by the European Medicines Agency (EMA) and the UK’s Medicines and Healthcare products Regulatory Agency (MHRA) [20].

Whilst intravitreal injections are an established mode of treatment in medical retina for multiple indications, adherence to and persistence with treatment varies significantly between indications [13]. The AGAIN programme of work sought to determine the acceptability of intravitreal injections for patients with GA, and the factors that may influence the acceptability of these treatments, using robust scientific methods.

The study consists of two related but distinct work packages. Work package 1 (WP1) began in Autumn 2020, an exploratory mixed-methods study—using structured (Likert-type scale) and semi-structured, open-ended questions—to explore the perspectives of 30 people living with GA on the acceptability of the new intravitreal injection treatments. Work package 2 (WP2) is a larger, multi-site, purely quantitative study that seeks to involve 180 individuals living with GA and quantify the proportion of patients who find the new treatments acceptable and correlate acceptability with ocular and demographic characteristics.

Acceptability, as defined by Sekhon and colleagues in their Theoretical Framework of Acceptability (TFA), is a “multi-faceted construct that reflects the extent to which people delivering or receiving a healthcare intervention consider it to be appropriate, based on anticipated or experienced cognitive and emotional responses to the intervention” [34]. Acceptability is a crucial and complex factor which can have implications for patients deciding to undergo a treatment, as well as adhering and persisting with it [24]. As such, assessment of prospective (i.e. anticipated) acceptability to patients should be a critical first step in the design, evaluation, and delivery of healthcare interventions [21]. The TFA underpinned our study design and explorations of how acceptable new intravitreal injection treatments are likely to be to people living with GA.

## Stage 1: Working with a PPI group to design a mixed-method questionnaire in Work Package 1 (WP1)

### Aim of involvement

Patient involvement was used to co-design a GA treatment information pack, and to shape the development of the questionnaire and topic guide underpinning the semi-structured interview in WP1. We also involved advisors in the review of a discrete choice experiment (DCE)-style task, where participants in WP1 would be asked to decide between two potential treatment scenarios and to explain the rationale for their choice.

### Method of involvement

Eight patient advisors with GA were identified and invited by authors CD and AG from their eye clinic in Brent, London, UK to be involved. All eight had expressed an interest in but were not eligible to take part in the current intravitreal treatment clinical trial for GA, meaning that they had some understanding of GA and the trials. (The corollary is that they were perhaps more likely to be more accepting of the treatment, given they had previously expressed interest in participating in the treatment trials.) The selection of advisors who were not eligible to participate in the GA treatment trials was considered a way to meaningfully involve individuals who had expressed interest in contributing to research but had not been able to participate in the treatment trials. We also did not wish to overburden patients who were already taking part in the trials themselves, even though they could feasibly have provided helpful, divergent perspectives. Patient advisors were all in their 70's or 80's; five were female, and three were male; four advisors were South Asian, three were white British, and one was East Asian. This broadly represents the ethnic diversity of the research site, where 64% of the population are from Black, Asian and minority ethnic groups. While aiming for balanced representation in terms of gender and ethnicity, we did not expressly consider other factors which could have influenced advisors’ perspectives, such as age, socioeconomic status, education level, or digital literacy. This is a clear limitation of our approach, and we could have been more systematic in ensuring maximal diversity among the patient advisors.

**Fig. 1:**
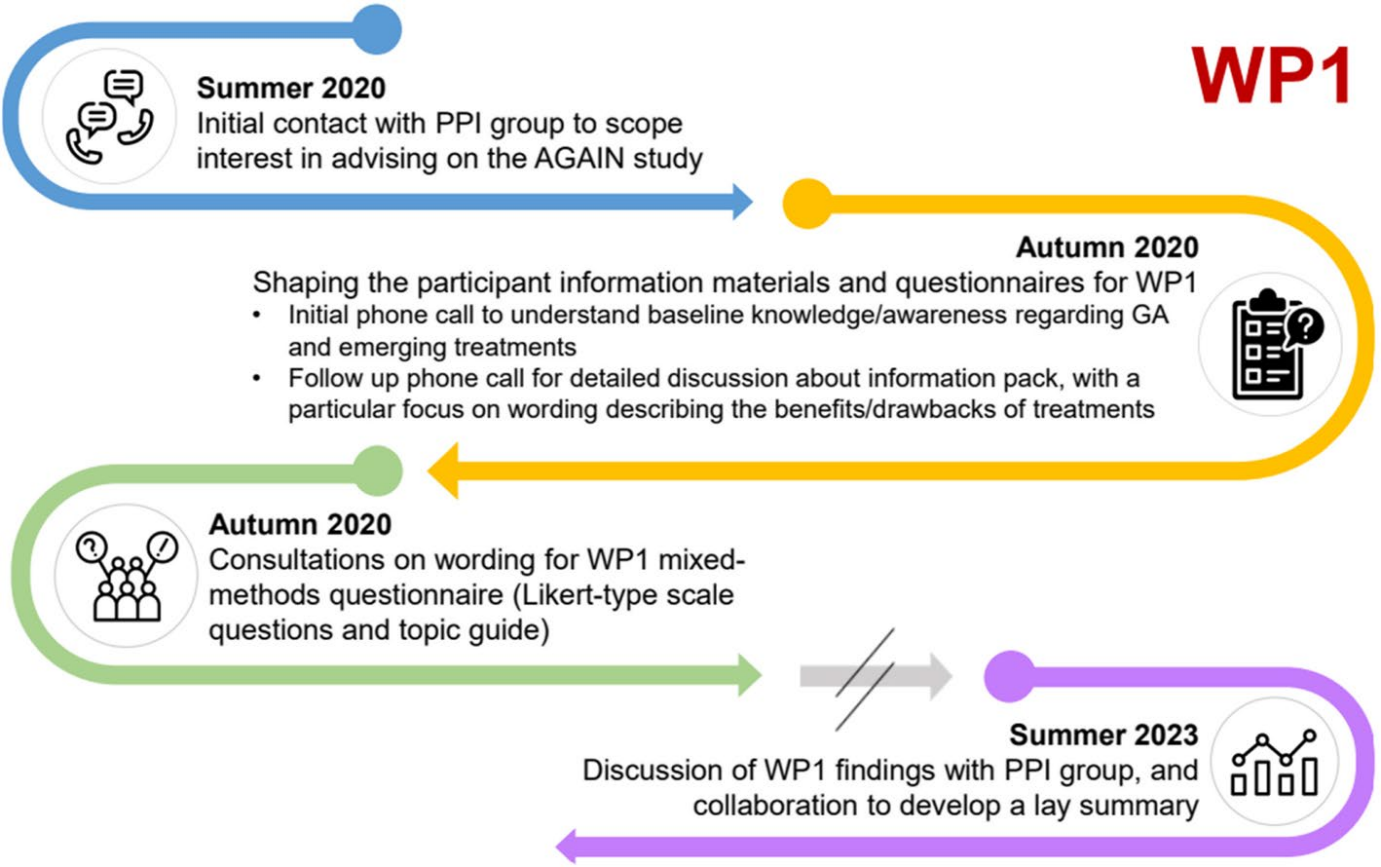
Summary of WP1 PPI process, showing the timing of notable PPI activities

Due to COVID restrictions, discussions with advisors took place by telephone, initially to explore their baseline level of knowledge of GA. These discussions were conducted in English, in one case in the presence of a family member who acted as an interpreter. Members of the research team conducting these discussions included ophthalmologists (authors AG and CD), and a research psychologist (author JE). It was made clear that these discussions were not formal research, and – in the case of discussion with ophthalmologists—would have no bearing on the patient advisors’ direct care. The aim was as far as possible to encourage advisors to openly share what first came to mind, without there being any ‘right or wrong’ answers. Based on these initial discussions (and our literature review), we formulated an initial draft questionnaire, consisting of Likert-type questions and a semi-structured interview topic guide, as well as an accessible information pack about the emerging intravitreal injection treatments for GA. After sending this to advisors, we conducted a second round of 1:1 telephone discussions, to seek views on the clarity of the questionnaire and information pack, and any suggestions for improvement. Finally, we sense-checked the final draft of questions and information materials with two advisors. A summary of the process of conducting PPI for WP1 is displayed in Fig. 1.

### Involvement results

Table 1 illustrates advisor comments recorded from the initial telephone discussions, and in response to queries and uncertainties regarding the questionnaire and information pack. As shown, the advisors made clear that the communication of the treatment benefits (slowing down vision loss by ‘up to 29%’) was considered abstract. This aligns with research suggesting that benefits or risks framed as percentages can be challenging to understand [29], without a ‘reference class’ translating the percentage into absolute terms [38]. Advisors questioned whether the rate of slowing would remain constant throughout the treatment course, and what evidence patients would have – in the absence of an improvement or stabilisation of vision – that the treatment was working effectively. Discussions also revealed that the advisors equated our non-interventional study on treatment acceptability with the (interventional) clinical trials of the new treatments. In all cases where this happened, the research team would clarify the independence of the present health services research activity (exploring patients’ views on the acceptability of treatments) from the biomedical and clinical research to develop GA treatments. Actions taken to address these issues raised by advisors, and to more broadly enhance the clarity and accessibility of the study materials, are displayed in Table 1.

**Table 1:** Examples of discussions with patient advisors and changes actioned in study design of WP1

Patient advisor feedback	Action taken in response to feedback
Patient advisors expressed a wish for a treatment that could stabilise or reverse vision loss from GA, and not just slow down the rate of vision loss	We reinforced at several points in the information sheet that GA treatments will not improve vision, only slow the deterioration
Notion of treatments slowing vision loss by 20–30% was considered too abstract and hard to understand	Information clarified with absolute example: “As a concrete example: without treatment, a person could be five years away from having to stop watching TV because of Geographic Atrophy. However, if they were having the treatment, then they could continue to watch TV for up to eighteen months longer.”
AGAIN study confused with clinical trials of intravitreal injection treatments	Information made clearer about our separation from clinical trial sponsor and investigators
The term “intravitreal injections” was deemed unnecessarily complicated	All mentions of “intravitreal injections” replaced with “eye injections”

**Fig. 2:**
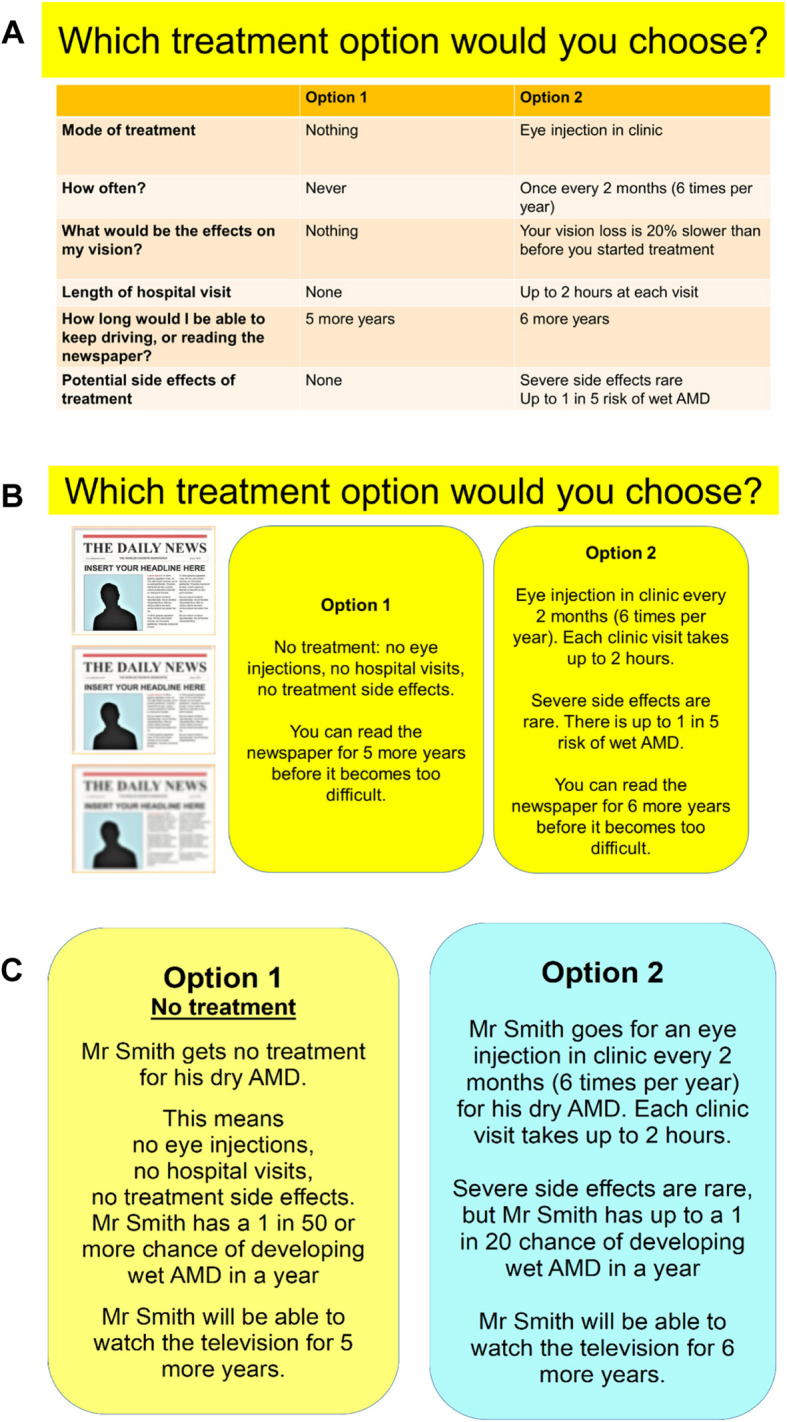
Scenarios for Discrete Choice Experiment (DCE)-style task, and how these evolved thanks to patient advisor input

Based on advisor feedback, the DCE materials evolved significantly. Firstly, instead of our initial idea of treatment scenarios shown side-by-side (Fig. 2A), almost all the advisors expressed a preference for a text block layout of information (Fig. 2B), stating that this is a “simple, gentle way to convey information”. In Fig. 2B, the participant was addressed directly as “You”, which created some confusion because some advisors could not identify with the scenario, for example if their GA and vision loss were too advanced and they would not benefit from treatment. For this reason, at the suggestion of one of the patient advisors, we adapted the treatment scenarios to focus on an imaginary patient, the generic “Mr Smith” (as shown in Fig. 2C). This aimed to address what one advisor described as “room for misunderstanding” and mitigate the risk of participants disengaging from the task if, for example, they felt that the five-year timeframe of living with vision adequate to watch TV was irrelevant for them. Furthermore, two advisors thought that the example of time remaining to read the newspaper (as in Figs. 2A and 2B) before it becomes too difficult might not be broadly applicable, as individuals may not read print newspapers; with television watching seen as a more universal activity, hence this change in the final version (Fig. 2C).

Having compiled the interview materials for WP1, including structured Likert-type scale questions, open-ended interview questions and the DCE-style task, we ran through all the materials with two patient advisors. One advisor responded that everything was clear, but we needed to be prepared for queries and potential misunderstandings about the treatments from participants. The other advisor suggested some additional prompts to the open-ended semi-structured interview questions, in case participants were unsure where to begin with their answer.

Following data collection and analysis, we drafted a lay summary of the study findings which was shared with the patient advisor group. This provided us with helpful insights in terms of how to disseminate the study results clearly [14]. For example, one advisor stated that we needed to highlight more prominently the finding (although this was expected) that acceptability of injections increased significantly as the time interval between injections increased.

## Stage 2: Adapting the theoretical framework of acceptability questionnaire in Work Package 2 (WP2)

Based on the TFA, Sekhon and colleagues have produced a pre-validated, generic questionnaire to assess the acceptability of any healthcare intervention, prospectively (i.e. as anticipated) or retrospectively (i.e. as experienced) from the perspective of patients or health professionals [35]. After adapting the questionnaire to the specific healthcare intervention of interest, Sekhon and colleagues recommend piloting with stakeholder advisors and the relevant patient population. This can help to ensure that questions are maximally comprehensible and answerable. In this section of the paper, we review background literature regarding involvement in questionnaires, especially patient reported outcome measures (PROMs), in order to contextualise the aims, methods and findings of PPI in the context of work package 2 (WP2) of the AGAIN study.

### The vital role of PPI in questionnaire development

There is now a relative consensus that PPI should play a key role in development of questionnaires and PROMs, although there is significant variation in the depth of this involvement [47]. In their scoping review, Wiering and colleagues conclude that the content validity of many PROMs, including commonly used PROMs such as the EQ-5D, could be improved through more thorough and meaningful involvement [30]. Staniszewska et al. [41] note the importance of representing the perspectives and voices of patients when designing PROMs, specifically with enhancing the wording and relevance of questionnaire items [6]. Indeed, an analysis by Taylor et al. [43] demonstrates that some of the most commonly used PROMs in ophthalmology require a higher reading level than the sixth grade (11–12 years of age) level recommended by the American Medical Association [44] and the US National Institutes of Health [25]. Therefore, such PROMs likely contain questions that are too difficult to be well understood. This highlights the importance of ensuring comprehensibility and readability when developing or adapting patient-facing questionnaires.

Feedback provided by patients and members of the public before questionnaires are finalised and formally deployed in health research studies can help to maximise the relevance, clarity and comprehensibility of questionnaire items. Wiering and colleagues [47] note that patients may be involved in the development of questionnaires in three main ways; determining important outcomes, generating items, and checking the questionnaire’s comprehensibility and content validity. (In the case of our study, the first and second stages were less salient because of using a pre-validated questionnaire [35]). Multiple studies discuss how patient and public involvement has helped in the development of research questionnaires [33, 39, 45]. However, literature documenting in detail how feedback from patients and the public was distilled and used to modify questionnaires is relatively scarce. One example is an article by Ayano Mes and colleagues [1], who show how patient contributor feedback led to changes in questionnaire instructions and item wording on an asthma medication questionnaire. Here, patient feedback led to changes on a questionnaire item for clarity, reducing the possibility of response error.

Some papers provide significant amounts of detail about patient, public or service user involvement in establishing the content and face validity of new questionnaires or PROMs. For example, Connell et al. [9] describe their process of interviewing service users about a new Recovering Quality of Life (ReQoL) measure for people with mental health difficulties [19]. Connell et al. conducted a full qualitative study exploring the questionnaire item relevance, ease of response, areas of potential ambiguity and potentially judgemental questionnaire items. The authors subsequently reshaped the ReQoL questionnaire; “some items were omitted, whilst others were reworded” [9], p1900). They also noted the importance of inviting feedback from a sample of service users representative of the target population, while highlighting the variation in the questionnaire items respondents find difficult or objectionable. In a similar level of detail, although presented within the context of PROM development rather than a full qualitative study, Patrick et al. [31] report methods for conducting cognitive interviews to explore patient understanding of items, instructions, and response options as a means of supporting content validity.

In ophthalmology, patient involvement in questionnaire development has often been documented in the earlier stages of determining key outcomes and questionnaire item generation. For example, patients with GA have been involved in the development of a core outcome set for GA clinical trials [22]. A systematic review of PROMs used in paediatric ophthalmology [42] identified several questionnaires that involved children and young people throughout the questionnaire development. These include the Child Amblyopia Treatment questionnaire (CAT-QoL) [7], which used cognitive debriefing interviews to explore the children’s ability to read and understand the questionnaire items. Carlton provides detail on how the wording of questionnaire items was changed in response to feedback from the children and when it was clear that they were struggling to understand certain terms (e.g. the concept of “frustration”).

### Aim of involvement

PPI was crucial in adapting the generic, pre-validated TFA questionnaire for use in our study.

While the studies discussed in the previous section concretely describe the changes made to questionnaires on the basis of PPI input, a key interest in this forthcoming section of the paper is reflecting on how to balance patient advisors’ different contributions. We aim to consider the complexities of reaching a consensus among varied points of view, while seeking to ensure that changes suggested by patient advisors do not annul the pre-validation of the source questionnaire. In light of our methodological protocol, there were fundamental constraints on how far the TFA questionnaire could be altered at a conceptual level. This meant that while the advice of patient advisors on the questionnaire was invaluable, the patient advisors were not embedded in the design of WP2 to the extent of this becoming a co-research or co-design endeavour.

### Method of involvement

**Fig. 3:**
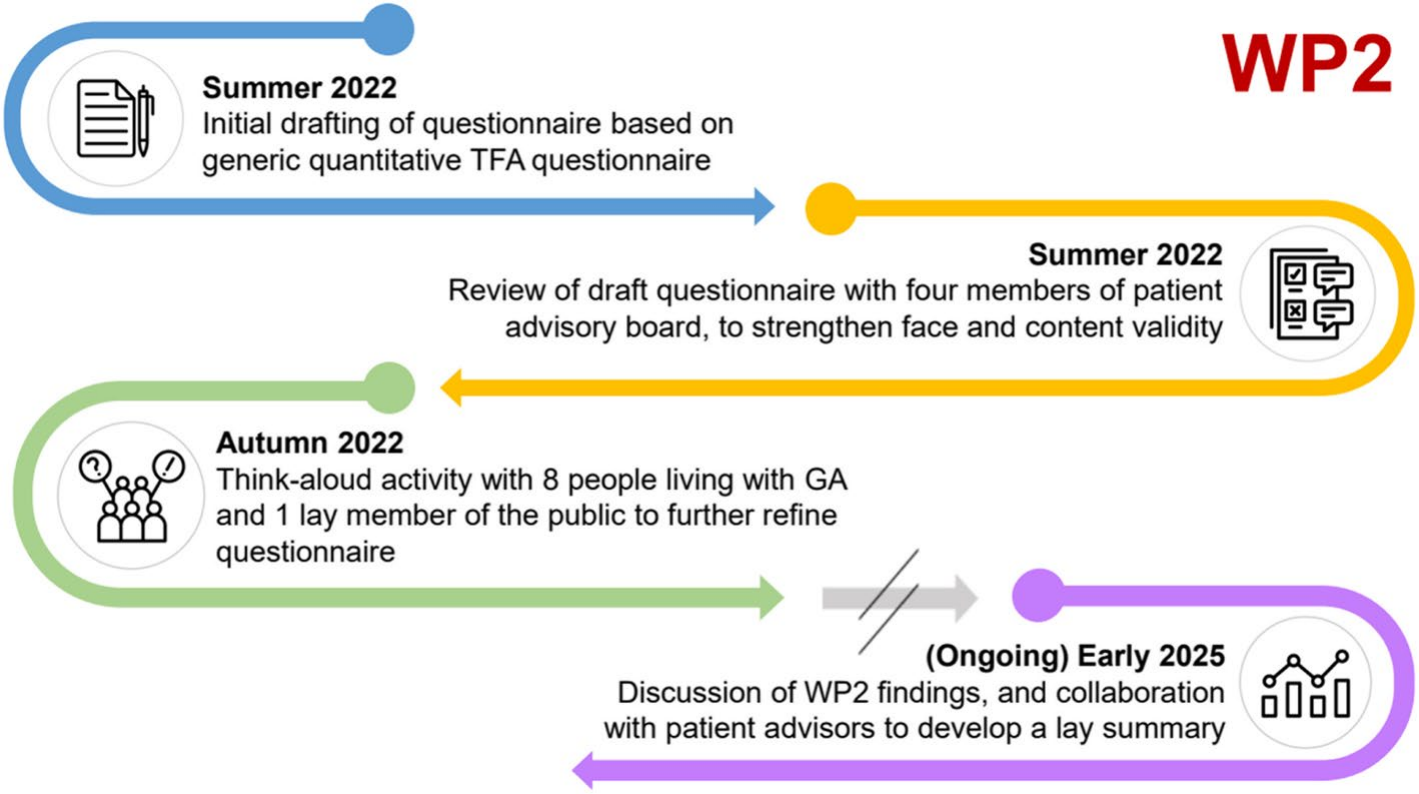
Summary of WP2 PPI process, showing the timing of notable PPI activities

A summary of the process of conducting PPI for WP2 is displayed in Fig. 3.

The principles of think-aloud studies were used in a PPI activity to adapt the pre-validated TFA questionnaire. Think-aloud studies are recommended as a method to elicit patient feedback on acceptability questionnaires Sekhon et al. [35]. They can involve elements of “cognitive interviewing” [31] or “cognitive debriefing” [7], whereby respondents explain their thinking and understanding regarding a question. Crucially, cognitive interviewing can mitigate against assumptions that all respondents to a questionnaire share a common understanding of “item content and intent” [31], p979). Think-aloud techniques have been used to elicit patient advisor views in other studies (e.g. [1, 3]. In contrast to a research study, the focus during our think-aloud activity was less on the content of the advisors’ responses, and more on how they interpreted and made sense of questionnaire items.

Our research team adapted the generic TFA questionnaire [35] to focus on GA treatment acceptability, using insights from WP1. We then held an initial round of think-aloud discussions with four patient advisors (who had been involved in the original advisory group for WP1, and who were still contactable and willing to be involved as advisors). We went through each question in turn, asking them if the question and response options were clear and if they had any feedback. Participants verbalised their feedback in real time, on a question-by-question basis. Notes were made by the research team, although discussions were not transcribed verbatim because this was a PPI exercise rather than a full ethically approved study.^2^ The questionnaire was then discussed with eight additional advisors who were *not* part of the core WP1 patient advisory group or hitherto involved in our study, in order to explore the comprehensibility and validity of the questionnaire with a group less familiar with treatment acceptability. Among this new group of advisors, the age ranged from people in their late 70's to late 90's affected by GA; four advisors were female and four were male; five advisors were white British, two were South Asian and one was other white. These new advisors were chosen from the Central Middlesex Hospital GA database, with a view to a balance of genders but not other socio-demographic factors; therefore, similar limitations as noted above regarding the WP1 advisory group also apply here.

As demonstrated in Fig. 3, WP2 is ongoing. We will continue to work with PPI advisors as we seek to disseminate and publicise the study findings to diverse audiences within the GA patient population, for example by producing accessible lay summaries of findings or using alternative media such as podcasts.

### Involvement results

Table 2 shows the original wording of the questionnaire, based on our adaptation of the TFA, the changes suggested by the patient advisors to improve clarity and validity, and the finalised questionnaire wording.

**Table 2:**
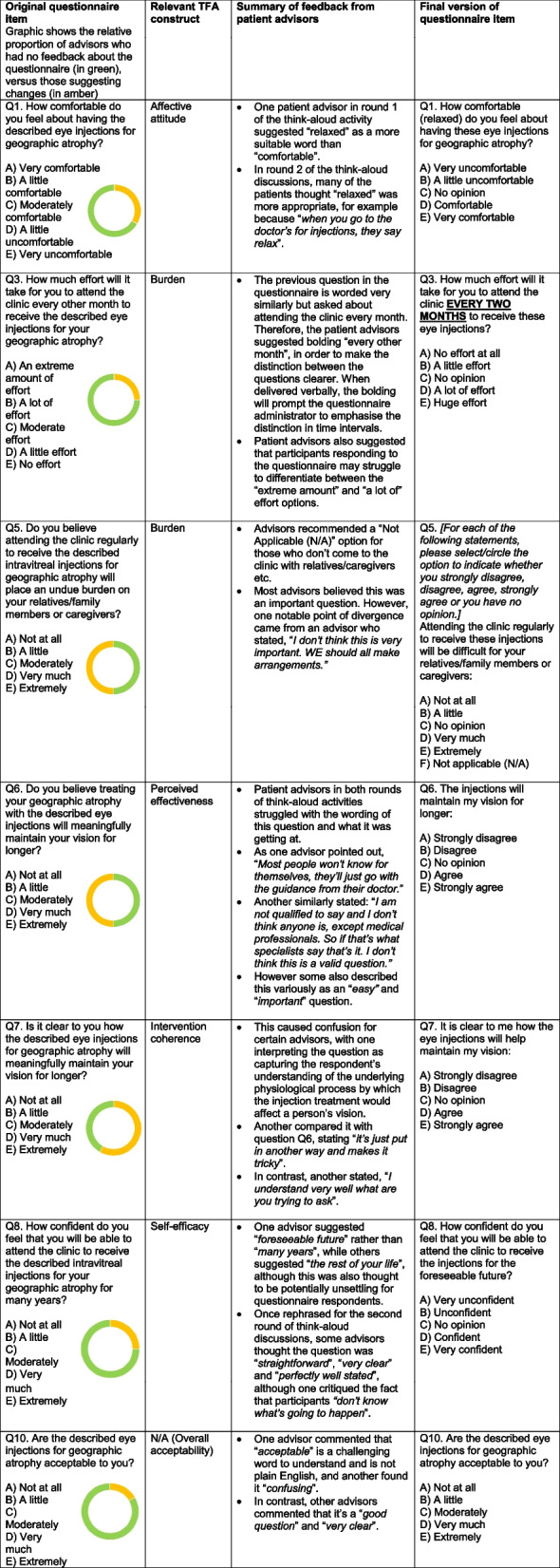
Item-tracking matrix, after Patrick et al. [31], based on think-aloud discussions. This summarises patient feedback during WP2 think-aloud activities and the resulting revisions made to the TFA-informed questionnaire

**Fig. 4:**
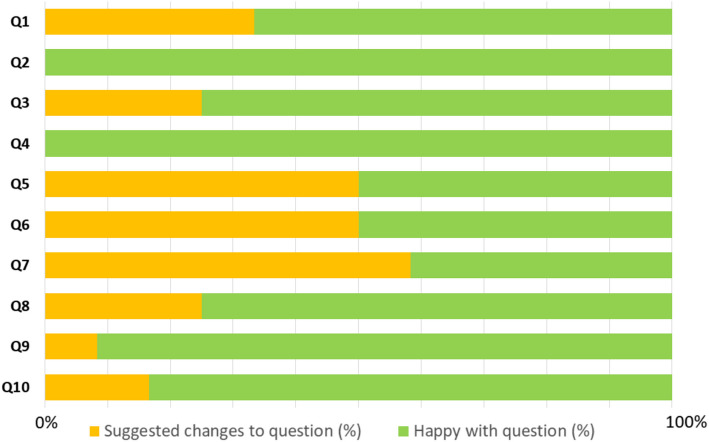
Bar chart highlighting the views of PPI advisors on each TFA-informed questionnaire item. The feedback is explored in further detail in Table 2

Table 2 highlights some of the feedback from patient advisors who participated in the two rounds of think-aloud discussions, illustrating the heterogeneity in patient advisors’ views on the conceptual and/or linguistic clarity of the different questionnaire items. Indeed, Table 2 demonstrates that for certain questions (e.g. Q8 and Q10), some patient advisors critiqued the question in terms of its lack of clarity, while others stated that the question wording was clear and straightforward. Feedback is summarised across all questionnaire items in Fig. 4; Fig. 4 provides a graphical overview of patient advisor feedback patterns, while Table 2 offers detailed examples of advisors’ feedback and changes made to the questionnaire. Overall, patient advisors commented that the questionnaire was easy to understand and complete, but there were a significant minority who felt that it was wordy or difficult to understand. One advisor stated that it is an “*easy questionnaire*”, provided that the Participant Information Sheet and the information provided to respondents about GA and the emerging treatments is sufficiently detailed.

## Discussion: balancing divergent perspectives—is consensus possible?

In terms of adapting the questionnaire in line with patient advisor feedback, we not only had to balance divergent perspectives among the different patient advisors but also think about how to take their advice on board, without moving so far from the generic TFA-based questionnaire that it would annul the pre-validation. In this section, we reflect on this challenge of listening to these divergent perspectives and their influence on the decisions made when developing the questionnaire.

As Table 2 demonstrates, for Q5, half of the patient advisors recommended changes, but these coalesced around the notion of adding a “Not applicable (N/A)” option for those without relatives/caregivers. This could be considered a ‘quick-win’, whereby modifying the questionnaire item/options was straightforward and did not risk introducing potential new ambiguities. In contrast, both Questions 6 and 7 were critiqued by half or slightly over half of the patient advisors respectively, but these critiques were more challenging to take on board. This is because the questions are specifically trying to tap into two of the constituent constructs of the TFA, Perceived effectiveness (Q6) and Intervention coherence (Q7), and we as a research team felt that the patient advisors’ critiques centred more around the concept itself than the wording of the question. We simplified the wording by framing the questions as agree/disagree statements, but felt that there were only limited adjustments that could be made to the TFA-based generic questionnaire in order to maintain these constructs [35]. We had concerns throughout the study that the need to adhere to the pre-validated TFA-based questionnaire limited the opportunities to fully incorporate advisors’ feedback. While we let the advisors know upfront that only limited changes to the wording were possible, and the reasoning behind this, it was a challenge to elicit feedback from advisors solely on the wording without moving into deeper discussion of the underlying concepts. This has been noted as an issue by other research groups integrating PPI into their studies. For example, Selman et al. [36] surveyed trial managers and public contributors involved with clinical trials, with one trial manager stating: “*They didn’t really change the design in terms of what our outcome measures were… because we were kind of stuck with using questionnaires that were already validated, even though they [public contributors] didn’t like them and sometimes it was quite frustrating that we couldn’t really make as many changes as we would have liked*”. This closely echoes our experience, encapsulating the dynamic we experienced of listening to the perspectives of patient advisors in our study and feeling unable to stray far from the wording of the pre-validated questionnaire. Otherwise, it is hard to find papers reporting on resolving differences in opinion, although some hint at this; for example, in their paper on PPI to develop a survey regarding prostate-specific antigen screening [5], the authors report that while the project group generally followed the recommendations of the PPI panel, some aspects of the survey “remained unchanged” with one specific question retained in its previous form “as a part of a standardized and validated instrument”. This similarly suggests that the limiting factor in taking patient and public contributors’ perspectives on board may be the necessity of adhering to the wording of validated questionnaires. This in turn underscores the importance of being transparent with advisors regarding the extent to which changes can be made to research instruments. Indeed, our experience suggests a need to be clearer with advisors about the scope of possible changes within the research design. For example, when inviting advisors to provide honest feedback on the clarity and comprehensibility of the question wording, we could have taken more care to ensure advisors’ understanding that the conceptual content of the questionnaire was essentially unalterable.

An issue which also warrants reflection is the unevenness with which patient advisors’ suggestions were taken on board, in the absence of a more formal, structured process for incorporating feedback via a consensus-based method. As shown in Table 2, there were more significant alterations to questionnaire items and responses for those questions where there was a greater preponderance of suggested changes from patient advisors. However, inevitably these changes involved listening to the suggestions of certain advisors and disregarding those of others, when we as the research team did not collectively agree that the suggested change represented an improvement in terms of comprehensibility or validity. Patient contributors to health research studies have noted this issue, for example feeling that “*researchers sometimes use PPI to back up their own views, but will query how representative it is if it disagrees with what they think. It’s vital to be clear how input will be handled*” [15]. Furthermore, as discussed above, suggested changes could not be actioned if the resulting question wording would stray too far from wording of the quantitative TFA questionnaire. In our study, we did not clarify in advance how different patient perspectives would be balanced and perhaps we could have pre-specified our approach to this more formally. At the same time, it appears that there is a dearth of theory and evidence in terms of ‘what works’ to balance divergent points of view, with the PPI literature discussing the importance of reaching a consensus (e.g. [17, 46]) without necessarily detailing the process of how that consensus is achieved [8]. As highlighted by Cary et al. [8], there is potential for neglecting attention to power dynamics and ensuring equity among different points of view. Furthermore, even with formal consensus-based methods such as Delphi studies, a judgement of ‘consensus’ when collecting qualitative feedback will inevitably involve subjectivity on the researchers’ part [2]. Nonetheless, we could have been more transparent at the outset of the think-aloud process about how patient advisors’ insights would be analysed and balanced, both against the views of the researchers and of other advisors.

## Conclusions and next steps: PPI throughout the AGAIN project lifecycle

In this article, we have aimed to provide a description and analysis of the process of embedding PPI into the design of the AGAIN study. We have highlighted the importance of listening to the insights of patients and those with lived experience in the early stages, ensuring that PPI remains continually integrated throughout the study lifecycle, and the beneficial impact PPI had on the study.

In terms of reflection and learnings for the future, we have sought to highlight not only the vital role PPI can play in improving the validity and comprehensibility of research materials, but also to demonstrate the challenges that may arise in terms of balancing divergent perspectives. It is also important to acknowledge that our study evolved in an ad hoc way due to funding cycles and in tandem, our PPI activities were undertaken in a largely ad hoc fashion, rather than using a carefully pre-planned, structured PPI strategy. For example, while we developed an information pack when inviting advisors to take part, which clarified that we would incorporate their advice and views into our study design, there was no formal agreement or terms-of-reference. Arguably in a larger research programme it may be more feasible to embed PPI in a formal, structured way from the very beginning. Larger programmes may also have more resources for PPI training early on in the project lifecycle, that can then upskill researchers on best practice in PPI. For example, we could have done more to involve patient advisors as co-researchers [4], which would require bespoke resources for training, in order to foster collaboration that also supports meaningful scientific contributions from co-researchers [23]. Additionally, we did not compensate advisors for their time, either financially or otherwise, but current guidance from the NIHR suggests it is good practice to pay PPI advisors wherever possible [26], an important learning for future PPI. Table 3 highlights these lessons learned and recommendations for other researchers to consider when conducting their own PPI activities.

The second work package (WP2) of the AGAIN study is still ongoing, and we will continue to consult with the patient advisors involved in our study as we move forward with data collection, analysis and dissemination of the findings.

**Table 3:** Key lessons and recommendations based on our PPI experience

• Our paper demonstrates the value of involving patients and the public in the design of research materials. However, we struggled with how far patient and public input should be taken on board when using questionnaires and research instruments that have already been previously validated. We wanted to take on board patients’ input to improve the clarity and comprehensibility of research materials, but were also concerned about ensuring fidelity to validated questionnaires we were using. We would recommend the development of strategies to reconcile a patient-led design with validated materials and procedures • Our PPI approach evolved organically, and began during the pilot phase of our study when there were limited resources in terms of time and personnel. Where possible, we recommend planning for and embedding PPI activities into research design from the start, and considering allocation of specific resources and funding for PPI efforts. This is especially important if seeking to involve patient advisors more extensively as co-researchers, and considering payment and reimbursement of advisors • In contrast to our more unstructured approach, there is likely to be value in pre-specifying an a priori method for how PPI feedback will be taken on board when designing the study materials and procedure, in order to have a robust strategy for incorporating divergent and sometimes conflicting subjective viewpoints	

## Supplementary Information

Supplementary Material 1.

## Data Availability

No datasets were generated or analysed during the current study.

## Footnotes

1 By pre-validated questionnaire, we mean a patient reported outcome measure that has already undergone a systematic process of content validity assessment, and assessment of comprehensibility, acceptability, relevance and answerability, but has not yet been formally validated in large samples (e.g. Prior et al., 2011).

2 All patient advisors gave verbal consent to participate in the discussions, and detailed notes were taken including of particularly notable quotes. However, discussions were not audio-recorded or transcribed, as this was not a research study *per se* and did not have, or require, ethical approval (in keeping with INVOLVE and university policy).
