# Supplementary material for: Implementing patient and public involvement (PPI) in eye research: reflections from developing a research study on Geographic Atrophy treatment acceptability
Source: Res Involv Engagem. 2025 Aug 1;11:90. doi: 10.1186/s40900-025-00747-7 (PMC12317502; doi:10.1186/s40900-025-00747-7)
Supplement: Supplementary file 1 — Supplementary Material 1. [file 40900_2025_747_MOESM1_ESM.docx]

# Appendix 1: GRIPP2 short form checklist

| **Section and topic** | **Item** | **Reported on page number** |
| --- | --- | --- |
| 1: Aim  Report the aim of PPIE in the study | To inform the design of a mixed-methods survey (in Work Package 1 – WP1) and a quantitative questionnaire based on the Theoretical Framework of Acceptability (Work Package 2 – WP2), including the information provided to participants and the research materials, such as the questionnaire items and responses. | WP1: p7  WP2: p16 |
| 2: Methods  Provide a clear description of the methods used for PPIE in the study | In WP1, eight patient advisors were involved in co-designing the information provided to participants, the survey questions (Likert-type scale questions and a semi-structured interview topic guide) and providing feedback on materials used in a Discrete Choice Experiment-style task.  In WP2, two rounds of think aloud activities were conducted (involving 4 of the patient advisors from WP1, and then 8 further patient advisors unfamiliar with the study) in order to elicit feedback on a quantitative questionnaire based on the Theoretical Framework of Acceptability. | WP1: p7-8  WP2: p16-17 |
| 3: Study results  Outcomes—Report the results of PPIE in the study, including both positive and negative outcomes | PPIE contributed to the study in the following ways.  For WP1:   - In initial one-to-one discussions, patient advisors helped us to understand their knowledge and awareness of Geographic Atrophy (GA) and their perspectives on the potential candidate treatments. - Helping to clarify the wording of the participant information sheet, including information about the treatments (on which participants’ judgements of prospective acceptability would be based). - Advising on the scenario content and presentation for the Discrete Choice Experiment (DCE)-style task. - Improving the comprehensibility and clarity of the Likert-type scale questions and topic guide. - Advising on a lay summary of the study findings and avenues for dissemination.   For WP2:   - Think-aloud discussions with 4 patient advisors (from the group contributing to WP1) - and further think-aloud discussions with 8 patient advisors not involved in WP1 - to improve the face and content validity of the questionnaire based on the Theoretical Framework of Acceptability (TFA). This helped to improve the comprehensibility and clarity of the questionnaire, although there were limits to how far patient advisors’ feedback could be actioned given the need to adhere to the wording of the pre-validated questionnaire. | p8-9  p8-9  p9-11  p12  p12  p18-21 |
| 4: Discussion and conclusions  Outcomes—Comment on the extent to which PPIE influenced the study overall. Describe positive and negative effects | PPIE has made an invaluable contribution to the AGAIN study so far. Particularly in the early stages of WP1, feedback from the patient advisors allowed us to ‘course-correct’ the design of our approach and research materials, and ensured the information we ended up issuing to participants about the new GA treatments was as clear and comprehensible as possible. In WP2, patient advisors’ contributions allowed us to improve the face and content validity of an acceptability questionnaire adapted from the generic TFA questionnaire.  While not necessarily a ‘negative’, in the article we reflect on the challenges of ensuring that all patient advisors’ points of view are taken on board, and considering how to find a balance between the advisors’ differing perspectives as well as the research team’s views.  While we built in PPIE from the start, our study is limited by the fact that the research programme evolved organically due to funding structures and there was not necessarily resource budgeted for PPIE training, or involvement of PPIE advisory group members as more active co-researchers. | p8-12 (WP1)  p18-21 (WP2)  p21-24  p24-25 |
| 5: Reflections/critical perspective  Comment critically on the study, reflecting on the things that went well and those that did not, so others can learn from this experience | On the basis of our experience, we believe that proactive involvement and engagement of patient advisors/contributors has a vital role to play in patient-centred research in ophthalmology and beyond. We have realised that financial and time resources are vital for PPIE to be carried out effectively, and funding structures should ensure that resources are apportioned for PPIE throughout the study; with such resources, we could have involved patient advisors more actively as co-researchers, for example involving them more centrally in the data collection and analysis processes. We have also reflected on the tension between listening to advisors’ suggestions and the limits imposed by pre-validated research instruments, and felt there is a potential gap in the literature and PPIE guidance in terms of how to handle this tension. | p.21-25 |
